# Effect of Cerenkov Radiation-Induced Photodynamic Therapy with ^18^F-FDG in an Intraperitoneal Xenograft Mouse Model of Ovarian Cancer

**DOI:** 10.3390/ijms22094934

**Published:** 2021-05-06

**Authors:** Yi-An Chen, Jia-Je Li, Syue-Liang Lin, Cheng-Hsiu Lu, Sain-Jhih Chiu, Fong-Shya Jeng, Chi-Wei Chang, Bang-Hung Yang, Ming-Cheng Chang, Chien-Chih Ke, Ren-Shyan Liu

**Affiliations:** 1 Institute of Clinical Medicine, National Yang Ming Chiao Tung University, Taipei 112, Taiwan; yachen0414@gmail.com; 2 Molecular and Genetic Imaging Core/Taiwan Mouse Clinic, National Comprehensive Mouse Phenotyping and Drug Testing Center, Taipei 112, Taiwan; rocket2350.be04@nycu.edu.tw (C.-H.L.); e2689@ym.edu.tw (S.-J.C.); jengfs@ym.edu.tw (F.-S.J.); 3 Department of Biomedical Imaging and Radiological Sciences, National Yang Ming Chiao Tung University, Taipei 112, Taiwan; d49620006@ym.edu.tw (J.-J.L.); SLR@nycu.edu.tw (S.-L.L.); bhyang@vghtpe.gov.tw (B.-H.Y.); 4 Department of Biotechnology and Laboratory Science in Medicine, National Yang Ming Chiao Tung University, Taipei 112, Taiwan; 5 Biomedical Engineering Research and Development Center Industrial, National Yang Ming Chiao Tung University, Taipei 112, Taiwan; 6 Industrial Ph.D Program of Biomedical Science and Engineering, National Yang Ming Chiao Tung University, Taipei 112, Taiwan; 7 National PET and Cyclotron Center (NPCC), Department of Nuclear Medicine, Taipei Veterans General Hospital, Taipei 112, Taiwan; cwchang@vghtpe.gov.tw; 8 Institute of Nuclear Energy Research, Atomic Energy Council, Executive Yuan, Taoyuan County 325, Taiwan; mcchang@iner.gov.tw; 9 Department of Medical Imaging and Radiological Sciences, Kaohsiung Medical University, Kaohsiung 807, Taiwan; ccke@kmu.edu.tw; 10 Drug Development and Value Creation Research Center, Kaohsiung Medical University, Kaohsiung 807, Taiwan; 11 Department of Medical Research, Kaohsiung Medical University Hospital, Kaohsiung 807, Taiwan; 12 Department of Nuclear Medicine, Cheng Hsin General Hospital, Taipei 112, Taiwan

**Keywords:** ovarian cancer, photodynamic therapy, Cerenkov radiation, ^18^F-FDG, photosensitizer

## Abstract

Ovarian cancer (OC) metastases frequently occur through peritoneal dissemination, and they contribute to difficulties in treatment. While photodynamic therapy (PDT) has the potential to treat OC, its use is often limited by tissue penetration depth and tumor selectivity. Herein, we combined Cerenkov radiation (CR) emitted by ^18^F-FDG accumulated in tumors as an internal light source and several photosensitizer (PS) candidates with matched absorption bands, including Verteporfin (VP), Chlorin e6 (Ce6) and 5′-Aminolevulinic acid (5′-ALA), to evaluate the anti-tumor efficacy. The in vitro effect of CR-induced PDT (CR-PDT) was evaluated using a cell viability assay, and the efficiency of PS was assessed by measuring the singlet oxygen production. An intraperitoneal ES2 OC mouse model was used for in vivo evaluation of CR-PDT. Positron emission tomography (PET) imaging and bioluminescence-based imaging were performed to monitor the biologic uptake of ^18^F-FDG and the therapeutic effect. The in vitro studies demonstrated Ce6 and VP to be more effective PSs for CR-PDT. Moreover, VP was more efficient in the generation of singlet oxygen and continued for a long time when exposed to fluoro-18 (^18^F). Combining CR emitted by ^18^F-FDG and VP treatment not only significantly suppressed tumor growth, but also prolonged median survival times compared to either monotherapy.

## 1. Introduction

Photodynamic therapy (PDT) is a minimally invasive form of therapy that has been clinically approved for treating non-oncological diseases as well as various types of cancers at the early stage [1,2]. Typically, light activates photosensitizers (PSs) to transform from the ground state to an excited state, producing reactive oxygen species (ROS) that cause cell damage. Since the light source utilized in clinical practices is external and commonly belongs to the visible light wavelength (400 to 800 nm), it exhibits limited tissue penetration (only up to 12 mm) within the body; therefore, it is not always effective when PDT is employed to deep-seated targets [3]. Cerenkov radiation (CR), which is based on a UV and blue light (250 to 600 nm), happens when charged particles generated from radioactive decay traveling in a dielectric medium with a velocity faster than the speed of light [4,5]. CR emitted from various medically relevant radioisotopes, such as ^18^F, ^11^C, ^68^Ga, ^64^Cu, ^15^O, ^131^I, etc., has been used not only in preclinical animal optical imaging equipment for Cerenkov luminescence imaging (CLI), but also served as an endogenous light source for PDT [6,7]. Recent studies have confirmed its feasibility; however, the efficiency of combining PSs needs to be further evaluated.

Porphyrin and chlorins-type structures constitute the largest group of PSs and have long been activated using red light in PDT owing to the presence of several Q-bands extending to the 630 nm region. However, there is a strong absorption band peaking at around 400 nm, known as the Soret band, suggesting that CR can serve as a light source to excite the PSs more efficiently. Based on the properties, Chlorin e6 (Ce6), 5′-Aminolevulinic acid (5′-ALA) and Verteporfin (VP), which have been approved by the FDA for anti-cancer applications, dermatologic indications or age-related macular degeneration, were thus were applied for CR-induced PDT (CR-PDT) against cancer in this study [1,8].

Ovarian cancer (OC) is one of the most common gynecologic malignancies and has the worst prognosis and the highest death rate, which can be mainly attributed to the majority of OC patients suffering from widespread metastasis and advanced disease when diagnosed [9]. The biology of OC differs from that of hematogenously metastasizing cancers in that no anatomical barrier prevents metastasis throughout the peritoneal cavity, suggesting that abdominal organs are vulnerable to adhesion and invasion. Once small clusters of OC cells are shed into the peritoneum from the primary tumor, numerous nodules colonize the intra-peritoneal organs to form secondary lesions. The main therapeutic strategy for OC is surgery followed by platinum/paclitaxel-based chemotherapy; however, drug resistance or difficulties with peritoneal micrometastasis eradiation eventually leads to treatment failure [10]. Although several studies have shown the potential of PDT with 5′-ALA and red/violet light for OC treatment, the lack of tumor selectivity and the limited tissue penetration of light have made it difficult to reach a satisfactory therapeutic effectiveness [11,12].

The most widely-used tracer in positron emission tomography (PET) is 2-deoxy-2-[F-18]fluoro-d-glucose, ^18^F-FDG, which accumulates in metabolically active cells and thereby differentiates malignant tumor from normal tissue [13]. Thus, ^18^F-FDG could be an ideal CR source in cancer cells to excite PSs with dominant Soret bands. In addition, we established an intraperitoneal xenograft mouse model of ovarian cancer using ES2 cell lines that developed into undifferentiated carcinoma and peritoneal dissemination, which is most common clinically [14]. Taken together, this study aimed to evaluate the efficiency of CR-PDT with ^18^F-FDG for OC treatment.

## 2. Results

### 2.1. In Vitro Effect of CR-Induced PDT with ^18^F-FDG

**Figure 1:**
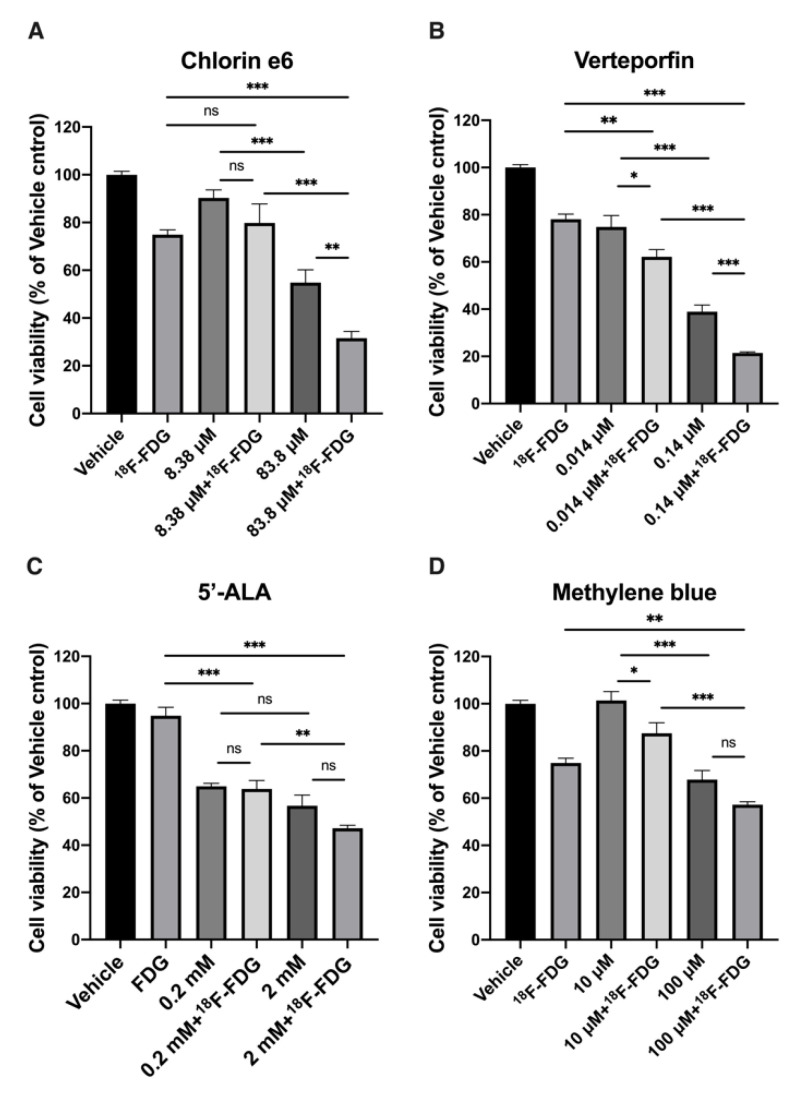
In vitro effect of CR-PDT with ^18^F-FDG. Cell viability assay comparing (**A**) Chlorin e6, (**B**) Verteporfin, (**C**) 5′-ALA and (**D**) methylene blue with and without treatment with ^18^F-FDG on ES2-luc cells. In each group, 3.7 MBq of ^18^F-FDG was used. Values are means ± SD (Each experiment was performed in triplicates and replicated 3X). * *p* < 0.05, ** *p* < 0.01, *** *p* < 0.001 and n.s., nonsignificant by Student’s *t*-test.

At first, the sensitivity of ovarian cell line-ES2 to ^18^F-FDG dose was determined (Figure S1). The reduction of cell viability was found in a dose-dependent manner and remained ~80% of the control group when ES2 cells incubated with 3.7 MBq of ^18^F-FDG. The dose was subsequently applied to in vitro experiments. We then evaluated the effect of CR-PDT on ES2 cells by combining ^18^F-FDG with the well-known PSs, 5′-ALA, methylene blue, Ce6 and VP, respectively. The concentrations of the PSs were chosen according to the rules that no dark toxicity could be observed in the cells, whereas the significant cytotoxicity would increase in the presence of light [15,16,17,18]. Two different concentrations of PSs (1 and 1/10 dilution) were incubated with ES2 cells followed by 3.7 MBq of ^18^F-FDG to examine the individual and combined effects in vitro (Figure 1). The results showed that all viabilities of the ES2 cells treated with ^18^F-FDG and higher PS concentrations were significantly deceased compared to either the ^18^F-FDG or higher PS concentration treatment alone group. Notably, the reduction of cell viability was dependent on the Ce6 or VP concentration under the same doses of ^18^F-FDG; higher concentrations of Ce6 or VP caused more cell death, to 31.5% and 21.4%, respectively (Figure 1A and B). Although the combination group of 5′-ALA or methylene blue also decreased cell viability to 47.5% and 57.2%, no significant combined effects were found when compared with cells treated with 5′-ALA or methylene blue alone (Figure 1C and D). The results indicated that Ce6 and VP were more effective PSs for CR-PDT in vitro. Furthermore, the IC_50_ (50% inhibition concentration) was determined for VP and Ce6 (Figure S2). Verteporfin was found to inhibit cell growth in a dose-dependent manner, and the IC_50_ was 0.21 µM. Interestingly, there was no reduction of cell viability at a lower concentration of Ce6; instead, cell growth was slightly increased, suggesting that Ce6 could induce cell proliferation.

### 2.2. Evaluation of ^18^F-Emitted CR-Induced Photoexcitation

**Figure 2:**
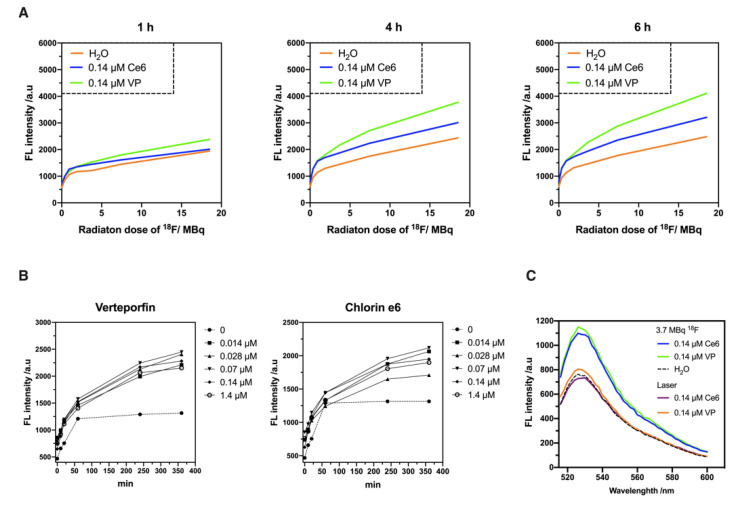
Evaluation of ^18^F-emitted CR-induced photoexcitation. In all experiments, SOSG, a fluorescent probe, was used for ^1^O_2_ detection. (**A**) Fluorescence spectra (λ_ex_ = 504 nm) were recorded when different doses of ^18^F were incubated with fixed concentrations of Chlorin e6 and Verteporfin, respectively, until 6 h. (**B**) Comparison of Chlorin e6 and Verteporfin with varied concentrations reacting with 3.7 MBq of ^18^F-FDG in FL intensity at 530 nm. (**C**) Comparison of the fluorescence spectra between CR and laser irradiation induced photoexcitation. For CR, 3.7 MBq of ^18^F-FDG was used to react with Chlorin e6 and Verteporfin. For laser irradiation, Chlorin e6 and Verteporfin were exposed under the laser pointer with emission wavelength of 650 ± 10 nm.

Among all ROS generated by the photoexcitation of PSs, singlet oxygen (^1^O_2_) is regarded as a highly effective killer of tumor cells. The lifetime of ^1^O_2_ is less than 0.04 μs and the radius of action is only approximately 0.02 μm in cells, thus contributing to the destruction of local tumors while reducing the risk of damage to surrounding normal tissue [19]. Herein, the efficiency of the PSs that applied in CR-PDT was evaluated by measuring the ^1^O_2_ production. The fluorescence (FL) intensity at 530 nm after exposure to an increasing dose of ^18^F was recorded, showing that the FL intensity was increased with increasing radiation doses and exposure time (Figure 2A). A comparison of the FL intensity between Ce6 and VP at the same concentration (0.14 μM) indicated that VP was more efficient in the generation of ^1^O_2_ and maintained for a long time. Despite some increase of FL intensity being observed in the mixture of fluoro-18 (^18^F) and the SOSG solution, which is the fluorescent probe for ^1^O_2_, both trend and intensity were much smaller than the fluorescent signal from the PS-containing solution (Figure S3). The ^18^F excited at 504 nm did not induce fluorescence emission as a check. Furthermore, the effects of the Ce6 and VP concentrations on the fluorescence performance were examined after exposure to a fixed radiation dose (3.7 MBq, as used in in cell viability tests). As shown in Figure 2B, the FL intensity increased with the exposure time and exhibited an upward trend in the presence of various concentrations of VP as well as Ce6 (ranging from 0.014 to 1.4 μM). However, there were no obvious trends of the FL intensity along with increasing concentration, and the FL signals actually declined at higher concentrations of Ce6 or VP, implying the self-quenching of the PSs occurred to limit ^1^O_2_ production (Figure S4). Despite this, the efficiency of VP was slightly higher than Ce6 under the CR derived from ^18^F.

Laser irradiation has been studied as a common light source for PDT; therefore, the efficiency of the photoexcitation was compared with that of ^18^F-emitted CR (Figure 2C). The fluorescence spectra of the photoexcitation of Ce6 and VP at the same concentration (0.14 μM) showed that the FL intensity of the ^18^F-emitted CR group was about 1.5-fold higher than that of the laser irradiation group, suggesting that the CR emitted by ^18^F was sufficient enough to induce ^1^O_2_ production. The results indicated that the potential of ^18^F-emitted CR as a light source to excite VP as well as Ce6 to generate ^1^O_2_ for cancer treatment.

### 2.3. Biologic Uptake and CLI of ^18^F-FDG in the ES2-Luc Xenograft Model

**Figure 3:**
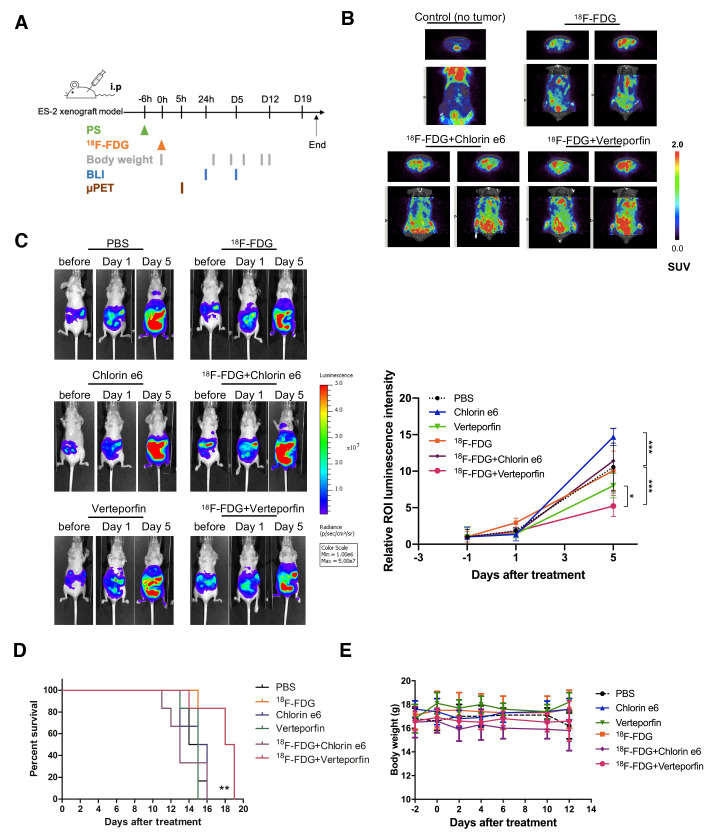
In vivo effect of CR-induced photodynamic therapy on ovarian cancer. (**A**) Schematic diagram for CR-PDT. Tumor bearing-mice were given intraperitoneal injection of Chlorin e6 or Verteporfin. Six hours later, mice were received 37 MBq of ^18^F-FDG intraperitoneally. (**B**) Biological uptake in tumor-bearing mice at 5 h after ^18^F-FDG injection. Representative images showing increased uptake in tumor-bearing mice but no difference among three groups that received ^18^F-FDG. (**C**) Evaluation of the effect of CR-PDT by optical bioluminescence image. Tumor burden in the peritoneal cavity was monitored at indicated time points. Representative bioluminescent images and quantification showing the combination of ^18^F-FDG-emitted CR and Verteporfin suppressed tumor growth in ES2-luc-bearing mice. Data represent mean ± SD (*n* = 5–6/group). * *p* < 0.05, ** *p* < 0.01, *** *p* < 0.001 by Student’s *t*-test. ** *p* = 0.0071, PBS versus ^18^F-FDG + Verteporfin; ** *p* = 0.016, Verteporfin versus ^18^F-FDG + Verteporfin; ** *p* = 0.004, ^18^F-FDG versus ^18^F-FDG + Verteporfin; ** *p* = 0.0099, ^18^F-FDG + Chlorin e6 versus ^18^F-FDG + Verteporfin; ** *p* = 0.028, PBS versus Chlorin e6 by Student’s *t*-test. (**D**) Kaplan–Meier survival curves representing the percentage of animals alive at the indicated time point after treatment. ** *p* = 0.0062, PBS versus ^18^F-FDG + Verteporfin; * *p* = 0.0197, Verteporfin versus ^18^F-FDG + Verteporfin; * *p* = 0.0154, ^18^F-FDG versus ^18^F-FDG + Verteporfin by Log-rank test. * *p* < 0.05, ** *p* < 0.01 (n = 6 mice per group). (**E**) The body weights of mice were recorded during the period of monitoring. Data are mean ± SD (n = 6/group).

Given that the PDT of combining ^18^F-FDG emitted CR with VP and Ce6 were efficient in killing ES2-luc cells, we next evaluated this therapeutic strategy using a clinically relevant model by implanting ES2-luc cells into nude mice intraperitoneally. These mice showed tumor cell dissemination and metastasis in the whole peritoneal cavity at an early time point and had a much shorter median survival time (less than 20 days) [14,20]. When the ES2-luc tumors reached approximately 2 × 10^8^ photons/sec (shown as the quantitative radiance value of IVIS imaging), the mice received an intraperitoneal injection of VP or Ce6 followed by ^18^F-FDG, respectively, and the treatment regimen was as shown in Figure 3A. To monitor the biologic uptake of ^18^F-FDG for CR-PDT, PET imaging and CLI imaging were used. The tumor-free mice showed ^18^F-FDG majorly accumulating in the heart and bladder followed by the gastrointestinal tract; thus, the physiological uptake image was regarded as the baseline image in this experiment (Figure 3B). A diffusely increased uptake was observed in the whole peritoneal cavity of all tumor-bearing mice at five hours after ^18^F-FDG injection, correlating with an increase in the SUVmean and SUVmax value of ^18^F-FDG. There was no difference among all tumor-bearing mice that had received ^18^F-FDG (Table 1). Furthermore, the Cerenkov luminescence (CL) of ^18^F-FDG was acquired using an IVIS 50 system, and a high correlation (R^2^ = 0.999) was observed between the luminescence intensities and the ^18^F activities, as shown as Figure S6A. The results showed that the CL emitted from the heart and whole peritoneal cavity were visually corresponded to the PET imaging and the signals from the ROI of the heart and peritoneal cavity were 8.1 × 10^3^ and 6.5 × 10^3^ p/s/cm^2^/sr, respectively (Figure S6B). These data demonstrated that the CR emitted by ^18^F-FDG accumulated in tumors has potential to be an endogenous light source for PDT.

### 2.4. In Vivo Effect of CR-Induced Photodynamic Therapy on Ovarian Cancer

Tumor growth in the peritoneal cavity was monitored by bioluminescence imaging (Figure 3C). There was no difference between the tumors treated with ^18^F-FDG alone and those treated with PBS; they both increased rapidly, revealing that the dosage of ^18^F-FDG (37 MBq) was not toxic to tumors. After receiving an intraperitoneal ^18^F-FDG injection, the post- and pre-luminescence ratios were not significantly different among all groups. On day five after treatment, the results showed slight inhibition of the tumor growth of ES2 in the mice receiving VP alone, while the VP and ^18^F-FDG combination treatment induced a significant inhibition compared with PBS treated controls (*p* = 0.071). The tumor burden in the mice receiving the combined treatment of VP and ^18^F-FDG was about two-fold less than that in the mice that received ^18^F-FDG alone. Moreover, the growth inhibition effect was evident in the combined VP- and ^18^F-FDG-treated mice than that of the VP-treated mice (*p* = 0.016). However, no anti-tumor effect was observed in the mice receiving either Ce6 alone or the combined Ce6 and ^18^F-FDG treatment. On the contrary, the tumor growth was drastically increased in the Ce6-treated mice, as assessed by in vivo imaging, compared to other treatment groups. The median survival time for each group of mice was recorded in Table S1, and a Kaplan–Meier plot was used to illustrate the results (Figure 3D). The median survival extended from 14.5 days for the PBS treated control to 18.5 days for the VP and ^18^F-FDG combination treatment group (*p* = 0.0062). A decrease in the median survival to 13 days for the Ce6 and ^18^F-FDG combination treatment group was also observed, suggesting the effect of the combination treatment overwhelmed the survival of the tested mice. The body weights of the treated mice were continuously monitored to investigate the systemic cytotoxicity of CR-PDT. There were no significant weight changes between groups at any time point post-treatment (Figure 3E). Taken together, the results showed that combining the CR emitted by ^18^F-FDG and VP not only suppressed tumor growth but also prolonged survival compared to either monotherapy.

## 3. Discussion

Our study demonstrated that using CR as internal light activated PS to generate ^1^O_2_ and led to cell death. These PSs have been proven to exert cytotoxicity in pre-clinical as well as clinical PDT trials for the treatment of cancer [3]. As far as CR-PDT is concerned, one of the primary considerations is to select a PS with CR luminescence that matches the absorption band. Since methylene blue (MB) is optimally excited at 660 nm, the maximal photodynamic effect could be found under a 660 nm laser exposure [15,21]. Cell viability experiments showed that the combination group of MB was less effective than the other PSs tested, indicating that CR light was not an ideal light source to activate MB (Figure 1). On the other hand, 5′-ALA is the precursor of protoporphyrin IX (PpIX) and has a Soret band peak at around 410 nm [22,23], suggesting that 5′-ALA should be suitable for CR-PDT. However, neither cytotoxicity at the indicated dose nor significant combined effect with ^18^F-FDG was found when treating the ES2 ovarian cell line. This result may have been due to very low intracellular PpIX accumulation in ES2 cells. Teshigawara et al. reported that the cytotoxicity of 5′-ALA in PDT is correlated with the level of intracellular PpIX, which might be attributed to the expression of PEPT1 (an ALA uptake transporter) and ABCG2 (a PpIX export transporter) [24]. They also found the ES2 cell lines express the highest level of ABCG2 and an undetectable expression of PEPT1. Thus, it is necessary to consider the properties of PS as well as characteristics of tumor cells prior to the application of PDT.

In our study, tumors treated with Ce6 plus ^18^F-FDG grew faster than those treated with ^18^F-FDG and PBS alone, suggesting that this combination did not inhibit tumor growth (Figure 3C). However, the effect of in vitro CR-PDT using Ce6 was comparable to using VP leading to cell death. Although this dose of Ce6 (40 mg/kg body weight) has exhibited remarkable anti-tumor effects in previous PDT studies [25,26], the concentration of Ce6 was diluted by ascites that usually occurred in our intraperitoneal OC mouse model; thus, it was insufficient to suppress tumor growth (Figure S5A). In addition, the efficiency of the ^1^O_2_ generation by Ce6 was less than that by VP under CR luminescence as shown in Figure 2B. Therefore, the inconsistency between the in vitro and in vivo CR-PDT was presumably derived from the dose of Ce6 used in the intraperitoneal administration. Moreover, the mice that received an intraperitoneal injection of Ce6 only showed a significant increase in tumor growth when compared with the other treatment groups. This result was in accordance with a slight increase in cell viability after incubating ES2 cells with a lower concentration of Ce6 (Figure S2).

In this study, the yield of ^1^O_2_ generation upon photoexcitation was measured using SOSG rather than other commonly used fluorescence probes, such as 1,3-diphenylisobenzofuran (DPBF) or 9,10-anthracenediyl-bis(methylene) dimalonic acid (ABDA), because SOSG shows a higher specificity and water solubility [27]. However, Liu et al. has reported that SOSG is not suitable for the detection of ^1^O_2_ in the existence of ionizing radiation due to various ROS production caused by the radiolysis of water, presenting a false-positive result [28]. Likewise, our data showed that the fluorescence intensity in the mixture of ^18^F and the SOSG solution was slightly increased with the increasing dose of ^18^F and exposure time. Nevertheless, the intensity was much smaller than the fluorescent signal from the PS-containing solution, indicating that the CR effect could still be distinguished by this approach. Although the most direct method for detection is to measure the luminescence of ^1^O_2_ at 1270 nm, this approach requires specialized equipment and is less sensitive to very small changes of ^1^O_2_ emission compared to fluorescence probes [29].

The disconnect between the success of preclinical animal studies and the outcomes of clinical trials has been attributed to mouse models of advanced disease or spontaneous metastasis, which have infrequently been used in such studies. Thus, our therapeutic strategy was demonstrated in a metastatic ovarian cancer model and compared with numerous studies for PDT. ES2 cell lines were selected owing to their highly metastatic properties (Figure S5) and also higher ^18^F-FDG uptake compared with several control tumor cell lines known to accumulate FDG [30]. The physiological uptake of ^18^F-FDG was clearly observed in the heart and gastrointestinal tract, as demonstrated in the PET imaging and CLI (Figure 3B and Figure S6B). Additionally, the results of the ex vivo CLI of ^18^F-FDG showed excessive accumulation in the reproductive tract. It was suggested that the implanted ES2 cells invaded the ovarian surface epithelium or metastasized through the peritoneal lymph node, which matched the characteristics of the intraperitoneal dissemination of ovarian cancer xenografts. We assumed that more ^18^F-FDG-avid lesions would be found not only in the reproductive tract but also in the kidneys and spleen, as well as other abdominal organs over a period of time. Notably, the significant therapeutic potential of CR-PDT was observed in the ES2 xenograft model. Considering the therapeutic effect of CR-PDT with ^18^F-FDG, our data demonstrated that a higher dose of ^18^F-FDG would generate more CR luminescence proportionately (Figure S6A) and induces more ^1^O_2_ production, indicating that a larger dose of ^18^F-FDG injection would be more effective than current strategies (Figure 2A). However, the permissible radiation dose is limited in humans and a previous study showed that mild hepatic cell damage could be observed in mice receiving 3.7 MBq of ^18^F-FDG intravenously [31,32]. It is for this reason that CR-PDT was performed using ^18^F-FDG less than 3.7 MBq in the mouse model.

Although our results showed the therapeutic potential of CR emitted by ^18^F-FDG induced PDT in combination with VP, there were also several limitations. First, the PSs involved in PDT exhibited some preferential accumulation in the tumors, but they were not tumor-exclusive. Targeted CR-PDT can be carried out by targeting the specific molecules that are not only overexpressed in tumor cells but are also critical for cell survival. For example, a nanoparticle consisting of VP and the Cetuximab, an epidermal growth factor receptor (EGFR)-blocking antibody, has the potential to kill ovarian cancer cells and could thus be used for the increasing treatment efficacy of CR-PDT [33]. Second, our therapeutic strategy consisted of a single injection of ^18^F-FDG and a PS. It is likely that repetitive CR-PDT could enhance the antitumor effect and benefit the survival of the treated mice dramatically. However, safety evaluations of the radiation dose, drug toxicity and repeated injections are warranted.

## 4. Materials and Methods

### 4.1. Cell Culture

The human ovarian cancer cell line ES2 stably expressing the luciferase gene (ES2-luc) was obtained from Dr. Chi-Mu Chuang (School of Medicine, National Yang Ming Chiao Tung University, Taipei, Taiwan) [34] and maintained in McCoy’s 5A (Modified) Medium (Catalog No.16600082, Thermo Fisher Scientific, Inc., Waltham, MA, USA), supplemented with 10% fetal bovine serum (Hyclone, Logan, UT, USA) in a humidified CO_2_ incubator at 37 °C. Cells were collected in a logarithmic growth phase and administrated into mice as soon as possible.

### 4.2. In Vitro CR-Induced Photodynamic Therapy

For PS preparation, 5′-Aminolevulinic acid (Sigma-Aldrich, St. Louis, MO, USA) and methylene blue (Sigma-Aldrich, St. Louis, MO, USA) were dissolved in de-ionized water (dH_2_O). Verteporfin (Sigma-Aldrich, St. Louis, MO, USA) and Chlorin e6 (Frontier Scientific, Logan, UT, USA) were dissolved in DMSO (Sigma-Aldrich, St. Louis, MO, USA). Subsequently, all PSs were added to the medium at the required concentration in indicated experiments. Five hundred cells were seeded in a 96-well cell culture plate and incubated for 24 h. Then, two different concentrations of PSs (1 and 1/10 dilution) were added into the culture medium and co-incubated for 4 h. After washing twice with PBS, the medium was replaced by 3.7 MBq of ^18^F-FDG-containing medium for 24 h. Relative cell viabilities were determined by a colorimetric method. CCK-8 reagent (Dojindo Molecular Technologies, Inc, Japan) was added into each well, and OD at 450 nm was measured using absorbance microplate reader (Sunrise, Tecan, Zürich, Switzerland) after incubation for 2 h at 37 °C. Cell viability (%) = (mean of OD of treatment group/mean of OD of control group) × 100. Results are shown as the mean ± SD of triplicate experiments.

### 4.3. Detection of Singlet Oxygen

The quantum yield of ^1^O_2_ generation upon photoexcitation was measured using Singlet Oxygen Sensor Green Reagent (SOSG, Molecular Probes, Eugene, OR, USA) as previously described [28]. In brief, ^18^F with activity ranging from 18.5 to 0.37 MBq (in 100 μL dH_2_O) was first thoroughly mixed with Ce6 or VP in black 96-well plate (Corning Costar, Tewksbury, MA, USA), followed by the addition of SOSG working solution and incubation until 24 h. Then, the fluorescence intensity (Excitation/Emission = 504 nm/516–600 nm) was measured using a microtiter plate reader (Tecan, Infinite M1000, Zürich, Switzerland). The emission peak for SOSG was at 530 nm and the slits of excitation and emission were 5 nm. For laser irradiation, a portable NIR commercial laser pointer (model LR6317, Chuiyung Technology Co., Ltd., ROC) with an emission wavelength of 650 ± 10 nm and ~200 mW of emitted power was used for irradiation of PSs, Ce6 or VP. After this, the mixture of PS and SOSG was prepared and transferred into a well, and the well was placed at a distance of 10.7 mm from the laser pointer for fixed periods of exposure time. Thereafter, the FL intensity was measured as described above. All experiments were performed in a dim environment.

### 4.4. Animal Model and In Vivo CR-PDT

Six-week-old female Balb/cAnNCrj-nu/nu mice (body weight, 16.9 ± 1.0 gm) were purchased from National Laboratory Animal Center (NLAC), NARLabs, Taiwan. Mice were group-housed (three to five animals per cage) in individually ventilated cages (IVC) systems. All animal procedures were carried out in strict accordance with the recommendations in the Guide for Care and Use of Laboratory Animals. The protocol was approved by the Committee on the Ethics of Animal Experiments of the National Yang Ming Chiao Tung University (IACUC number: 1081007r, permission date: 18 October 2019). To establish the xenografts, 2 × 10^6^ ES2-luc cells in 150 μL phosphate-buffered saline (PBS, Sigma-Aldrich, St. Louis, MO, USA) were injected intraperitoneally into the animal’s right lower abdominal quadrant. A therapeutic efficacy study was conducted at 6 d after cell injection. Tumor-bearing mice were randomized into six groups of six animals per group for the following treatments: (1) PBS (control), (2) 37 MBq of ^18^F-FDG injection only (^18^F-FDG), (3) Ce6 (40 mg/kg body weight), (4) VP (45 mg/kg body weight), (5) Ce6 followed by 37 MBq of ^18^F-FDG and (6) VP followed by 37 MBq of ^18^F-FDG. For intraperitoneal injection of PS, VP and Ce6 stock solutions were diluted with Tween 80 (Sigma-Aldrich, St. Louis, MO, USA) and PBS (ratio = 20:10:70) to achieve a final concentration of 10 mg/mL. All mice were kept fasting overnight and injected ^18^F-FDG intraperitoneally at 6 h after administration of PS and weighed to evaluate the systemic toxicity every two days.

### 4.5. MicroPET Imaging and Imaging Analysis

To confirm biological uptake of ^18^F-FDG, each mouse was imaged under anesthesia with 1 to 1.5% isoflurane using Triumph PET/SPECT/CT imaging scanner after injection (Gamma Medica-Ideas, CA, USA) at 5 h after 37 MBq of ^18^F-FDG injection. Additional CT scans are acquired after PET acquisition for anatomical localization. The PET images dataset was then reconstructed using the ordered-subset expectation maximization algorithm with standard-mode parameters and 2D maximum likelihood expectation maximization algorithm. Subsequently, the regional retention and uptake of ^18^F-FDG were processed and analyzed with AMIDE software. The value was reported as standardized uptake values (SUV) which represented the mean activity values for peritoneal cavity, normalized to the injected dose per body weight of each individual animal.

### 4.6. In Vivo Bioluminescence Image

The treatment effects were evaluated with relative bioluminescence measured before and at day 1 and day 5 after treatment. Mice were anaesthetized with isoflurane and intraperitoneally injected D-luciferin (150 mg/kg body weight). After 15 min, mice were placed in the chamber and then photo counts were acquired for 5 min by a bioluminescence imaging system (Perkin Elmer, Waltham, MA, USA). Region of interest (ROI) selection and signal quantification were performed using living image software 3.2 (IVIS 50 Imaging System, Perkin Elmer, Waltham, MA, USA). The bioluminescent signal was reported as the mean photons/s/centimeter^2^/steradian (photon/s/cm^2^/sr), represented by a pseudo-color photo count and laid over the photographic image, displaying both bioluminescence intensity as well as the mice anatomy.

### 4.7. Statistical Analysis

GraphPad Prism v.7.0 software (GraphPad, San Diego, CA, USA) was used to perform student’s *t*-test, one-way analysis of variance (ANOVA) with Tukey’s test correction and Kaplan–Meir survival curve. A *p* value of less than 0.05 was considered statistically significant.

## 5. Conclusions

Our study has provided in vitro and in vivo evidence suggesting that PSs with matched absorption spectra could be excited by ^18^F-FDG to induce cell death, thereby suppressing tumor growth and prolonging survival times. Furthermore, we found that VP exhibited better ^1^O_2_ generation efficiency, suggesting that CR-induced photoexcitation of VP could achieve the effective PDT in tumor cells. These results highlighted the beneficial effects of CR-PDT in OC treatment, reassessed well-known PSs, and could further facilitate the development of more specific radioactive probes with higher CR efficiency against cancers.

## Figures and Tables

**Table 1:** Biological uptake in ES2 xenograft models at 5 h after [^18^F]FDG injection.

Animal Groups	SUVmean	SUVmax
Control (no tumor)	6.71 ± 0.32	6.25 ± 1.49
[^18^F]FDG	7.49 ± 0.54	8.52 ± 1.78
[^18^F]FDG + Verteporfin	9.98 ± 3.10	7.98 ± 3.68
[^18^F]FDG + Chlorin e6	9.61 ± 5.69	8.91 ± 2.11
		*N* = 3

## Data Availability

Not applicable.

## Supplementary Materials

The following are available online at https://www.mdpi.com/article/10.3390/ijms22094934/s1, Figure S1: The sensitivity of ovarian cell line ES2-luc to ^18^F-FDG dose, Figure S2: IC_50_ of methylene blue, Chlorin e6 and Verteporfin, Figure S3: The radiation exposure induced fluorescence emission of the SOSG probe, Figure S4: Comparison of Chlorin e6 and Verteporfin with varied concentrations (0.014–83.8 μM) reacting with 3.7 MBq of ^18^F-FDG in fluorescence intensity at 530 nm, Figure S5: Gross pathology of xenograft tumors, Figure S6: In vitro, in vivo and ex vivo Cerenkov luminescence imaging of ^18^F, Table S1: Median survival time for each group after treatment.
