# Supplementary material for: Effect of Cerenkov Radiation-Induced Photodynamic Therapy with 18F-FDG in an Intraperitoneal Xenograft Mouse Model of Ovarian Cancer
Source: Int J Mol Sci. 2021 May 6;22(9):4934. doi: 10.3390/ijms22094934 (PMC8125334; doi:10.3390/ijms22094934)
Supplement: Supplementary file 1 [file ijms-22-04934-s001.zip › ijms-1177322 - SI.pdf]

Cerenkov Radiation Induced Photodynamic Therapy with ^{18}F-FDG for Disseminated Intraperitoneal Ovarian Cancer

Yi-An Chen[1, 2], Jia-Je Li[3], Syue-Liang Lin[3, 4, 5], Cheng-Hsiu Lu[2, 6], Sain-Jhih Chiu[2], Fong-Shya Jeng[2], Chi-Wei Chang[7], Bang-Hung Yang[3, 7], Ming-Cheng Chang[8] and Ren-Shyan Liu[1, 2, 6, 9, *]

1. Institute of Clinical Medicine, National Yang Ming Chiao Tung University, Taipei 112, Taiwan, ROC

2. Molecular and Genetic Imaging Core/Taiwan Mouse Clinic, National Comprehensive Mouse Phenotyping and Drug Testing Center, Taipei 112, Taiwan, ROC

3. Department of Biomedical Imaging and Radiological Sciences, National Yang Ming Chiao Tung University, Taipei 112, Taiwan, ROC

4. Department of Biotechnology and Laboratory Science in Medicine, National Yang Ming Chiao Tung University, Taipei 112, Taiwan, ROC

5. Biomedical Engineering Research and Development Center Industrial, National Yang Ming Chiao Tung University, Taipei 112, Taiwan, ROC

6. Industrial Ph.D Program of Biomedical Science and Engineering, National Yang Ming Chiao Tung University, Taipei 112, Taiwan, ROC

7. National PET and Cyclotron Center (NPCC), Department of Nuclear Medicine, Taipei Veterans General Hospital, Taipei 112, Taiwan, ROC

8. Institute of Nuclear Energy Research, Atomic Energy Council, Executive Yuan, Taoyuan County 325, Taiwan, ROC

9. Department of Nuclear Medicine, Cheng Hsin General Hospital, Taipei 112, Taiwan, ROC

* Correspondence: rsliu@vghtpe.gov.tw; Tel: 886-2-28757301

Supplementary Information Index

S1) The sensitivity of ovarian cell line ES2-luc to ^{18}F-FDG dose

S2) IC_{50} of methylene blue, Chlorin e6 and Verteporfin

S3) The radiation exposure induced fluorescence emission of the SOSG probe

S4) Comparison of the fluorescence intensity at 530 nm between Chlorin e6 and Verteporfin with varied concentrations (0.014-83.8 μM) reacting with 3.7 MBq of ^{18}F-FDG

S5) *In vitro, in vivo* and *ex vivo* Cerenkov luminescence imaging of ^{18}F

S6) Gross pathology of ES2-luc xenograft tumors

Table S1) Median survival time for each group after treatment

Materials and methods

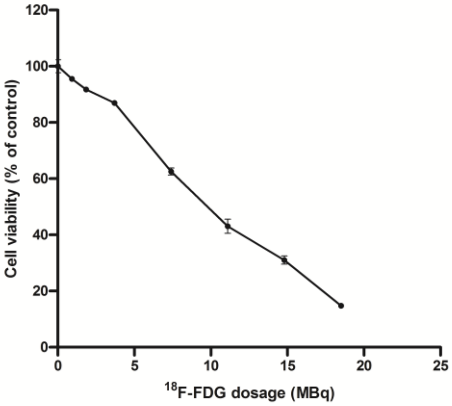

Supplementary figure S1. The sensitivity of ovarian cell line ES2-luc to ^{18}F-FDG dose. Cell viability of ES2-luc cells treated with serial radioactivity of ^{18}F (18.5, 14.8, 11.1, 7.4, 3.7, 1.85 and 0.93 MBq). Cells without treatment served as a control group. Values are means ± SD (experiments for each group were run in triplicates and replicated 3X).

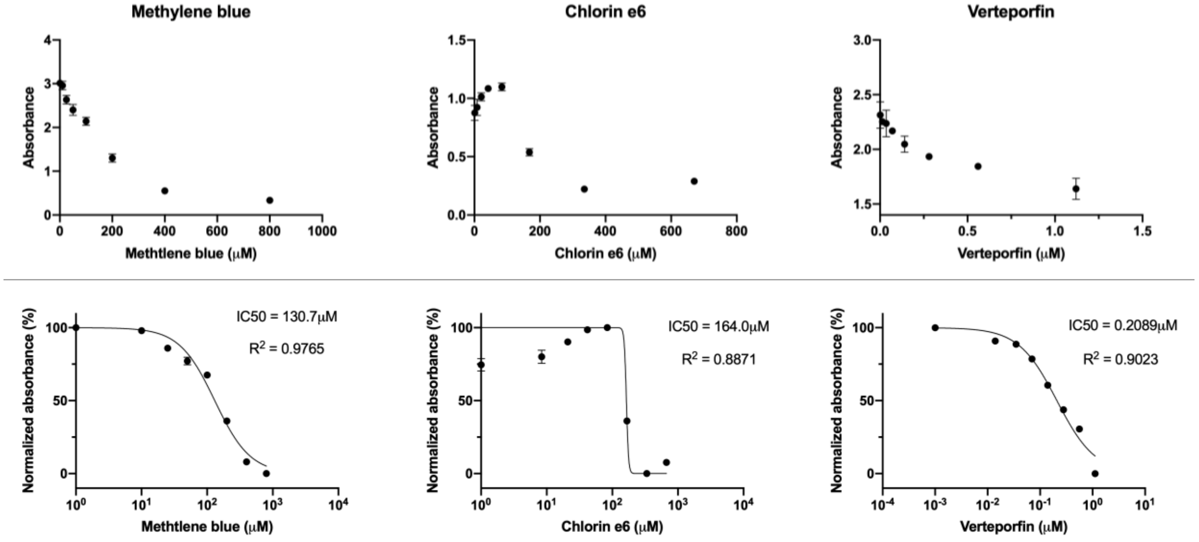

Supplementary figure S2. IC_{50} of methylene blue, Chlorin e6 and Verteporfin. ES2-luc cell line was treated with indicated concentration of methylene blue, Chlorin e6 and Verteporfin respectively for 48 h. Cell viability was assessed by CCK-8 assays. All data were normalized to cells without treatment. IC_{50} of methylene blue, chlorin e6 and verteporfin were calculated using GraphPad Prism software. Data are presented as mean ± SD.

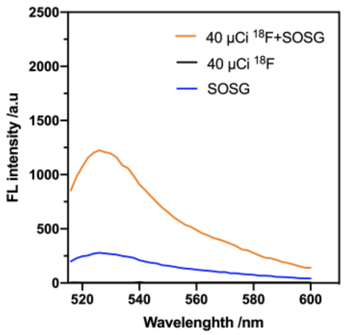

Supplementary figure S3. The radiation exposure induced fluorescence emission of the SOSG probe. Fluorescence spectra (λ_{ex}= 504 nm) of SOSG solutions with and without 40 µCi of ^{18}F treatment. Fluoro-18 excited at 504 nm did not induce fluorescence emission. The radiation exposure induced fluorescence emission of the SOSG probe.

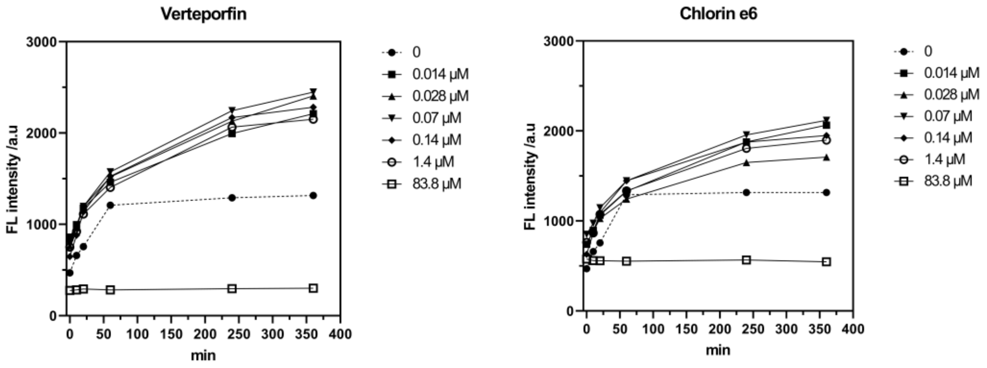

Supplementary figure S4. Comparison of the fluorescence intensity at 530 nm between Chlorin e6 and Verteporfin with varied concentrations (0.014-83.8 µM) reacting with 3.7 MBq of ^{18}F-FDG

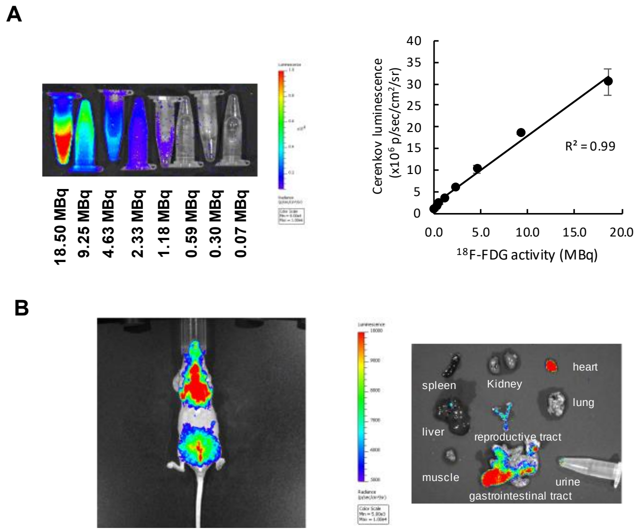

Supplementary figure S5. *In vitro*, *in vivo* and *ex vivo* Cerenkov luminescence imaging of ^{18}F. (A) Left panel: CLI of ^{18}F with the doses ranging from 10.07 to 18.5 MBq in Eppendorf tubes was performed using an IVIS 50 luminescence imaging system. Right panel: The CL signals of ^{18}F in each tube (shown as p/s/cm^2/sr) were linearly correlated with the radioactivity. (B) Left panel: In vivo CLI at 5 hours after receiving ^{18}F-FDG. Right panel: Abdominal organs were obtained to perform ex vivo CLI.

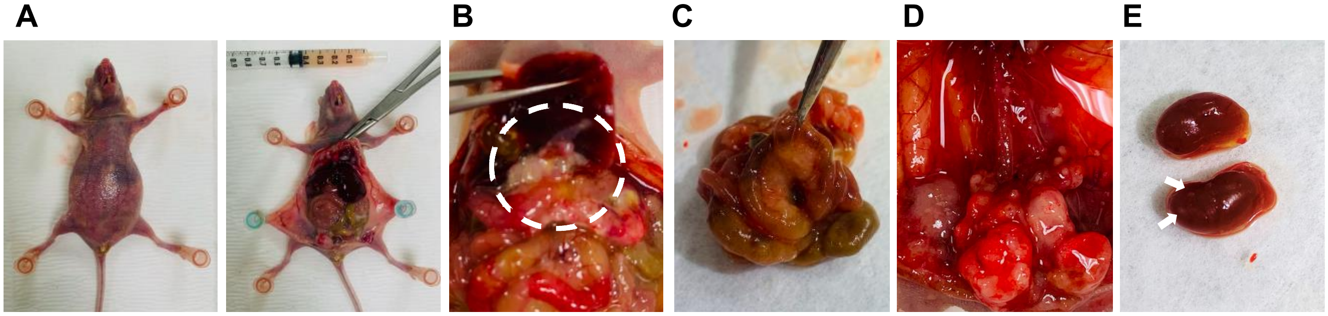

Supplementary figure S6. Gross pathology of xenograft tumors. The Abdominal organs were taken from ES2-luc xenograft mouse model and metastases were observe as shown by photos. (A) Highly cellular and milky ascites of ES2-luc. (B) Thickened omentum adheres to surrounding organs (white circle). (C) Mesenteric metastasis. (D) Metastasis on peritoneum. (E) Renal metastasis (white arrows).

Animal groups	Median survival time after treatment (days)
PBS	14.5
Verteporfin	15.0
Chlorin e6	15.5
[^{18}F]FDG	15.0
[^{18}F]FDG+Verteporfin	18.5
[^{18}F]FDG+Chlorin e6	13.0

Supplementary Table S1. Median survival time for each group after treatment.

MATERIALS AND METHODS

Cell Viability Assay

To evaluate the sensitivity of ES2-luc cells to ^{18}F-FDG dose, ES2-luc cells were seeded in a 96-well cell culture plate at the density of 5x10^3 cells/well and incubated with serial dilutions of ^{18}F-FDG (the dose of ^{18}F-FDG was 18.5, 14.8, 11.1, 7.4, 3.7, 1.85 and 0.93 MBq respectively in culture medium). Plates were then incubated 24 hours at 37 °C under 5% CO2. Relative cell viabilities were determined by a colorimetric method. CCK-8 reagent (Dojindo Molecular Technologies, Inc) was added into each well, and OD at 450 nm was measured using absorbance microplate reader (Sunrise, Tecan) after incubation for 2 h at 37 °C. Cell viability (%) = (mean of OD of treatment group/mean of OD of control group) x 100. Results are the mean ± standard deviation of triplicate experiments. For photosensitizer preparation, 5'-Aminolevulinic acid (Sigma-Aldrich, USA) and methylene blue (Sigma-Aldrich, USA) were dissolved in de-ionized water (dH_2O). Verteporfin (Sigma-Aldrich, USA) and Chlorin e6 (Frontier Scientific) were dissolved in DMSO. Subsequently, all photosensitizers were added to the medium at the required concentration in indicated experiments. IC_{50} values were calculated from a log([drug]) versus normalized response curve fit using GraphPad Prism version 7.0 for Mac (GraphPad Software).

Cerenkov luminescence imaging (CLI) *in vitro* and *in vivo*

To assess the correlation of CLI signals and ^{18}F activity *in vitro*, ^{18}F was serially diluted into 18.5, 9.25, 4.23, 2.33, 1.18, 0.59, 0.3 and 0.07 MBq in 200 µl saline in Eppendorf tubes. CLI was carried out using an IVIS 50 imaging system with a luminescence imaging setting of binning: 8, FOV: 12, f-stop: 1, exposure time: 300 s. The signal of ^{18}F-emitted CL was analyzed using Living Imaging Software and shown in p/s/cm^2/sr. For *in vivo* CLI, the whole peritoneal cavity of tumor-bearing mouse and related organs were observed at 3 h after intraperitoneal injection of ^{18}F-FDG. The setting and CL signals were performed and analyzed as described above.
